# A Microwave Microfluidic Sensor Based on a Dual-Mode Resonator for Dual-Sensing Applications

**DOI:** 10.3390/s17122713

**Published:** 2017-11-24

**Authors:** Nikolina Jankovic, Vasa Radonic

**Affiliations:** BioSense Institute—Research Institute for Information Technologies in Biosystems, Novi Sad 21000, Serbia; nikolina@biosense.rs

**Keywords:** microwave sensor, dual-mode resonator, microstrip, microfluidics, low-temperature co-fired ceramics

## Abstract

In this paper, we propose a novel microwave microfluidic sensor with dual-sensing capability. The sensor is based on a dual-mode resonator that consists of a folded microstrip line loaded with interdigital lines and a stub at the plane of symmetry. Due to the specific configuration, the resonator exhibits two entirely independent resonant modes, which allows simultaneous sensing of two fluids using a resonance shift method. The sensor is designed in a multilayer configuration with the proposed resonator and two separated microfluidic channels—one intertwined with the interdigital lines and the other positioned below the stub. The circuit has been fabricated using low-temperature co-fired ceramics technology and its performance was verified through the measurement of its responses for different fluids in the microfluidic channels. The results confirm the dual-sensing capability with zero mutual influence as well as good overall performance. Besides an excellent potential for dual-sensing applications, the proposed sensor is a good candidate for application in mixing fluids and cell counting.

## 1. Introduction

Microwave sensors have attracted considerable attention in the last decade since they operate in the range of frequencies 0.3–30 GHz, which are non-destructive and safe, yet having excellent detection potential even in the case of small sample volume. Also, microwave structures can be readily implemented in the integrated circuits, which have become a favourable platform due to their constantly decreasing cost. As such, microwave sensors have found numerous applications including permittivity sensing [1,2,3,4,5,6,7,8,9,10,11,12,13,14,15], strain and displacement sensing [16,17,18,19,20], detection of biomolecules [21], glucose monitoring [22,23], dielectric spectroscopy for food quality control [24,25], gas sensing [26,27,28], and concentration measurements of liquid solutions [29].

The operating principles of the proposed microwave sensors predominantly rely on the resonance concept [4,5,6,7,9,10,12,14,15,16,19,20,21,23,29], however there are also structures based on transmission line [1,2,18,26,27,28] and capacitive methods [13,17,22], as well as on more peculiar phenomena such as energy tunneling [3,11] and metasurfaces [8]. Besides split ring resonators (SRR) [4,9,14,15,16,19,20,21], which have been widely exploited in resonant-type sensors, there have also been proposed sensors with cavity [6,29] and stepped impedance resonators (SIR) [5,7]. As for the transmission line-based sensors, the majority of them have been realized using the substrate integrated waveguide (SIW) [27], coplanar waveguide (CPW) [18,26,28], microstrip with electromagnetic bandgap (EBG) structures [2] and microstrip differential lines [1], whilst capacitive sensors have been realized as a gap [13], plate [17] or interdigital [22] capacitor in microstrip configurations.

Recently, microwave sensors have been combined with microfluidics—a technology of manipulation of small quantities of fluids, which has found various biological, environmental, and sensing applications [30,31,32,33,34,35,36,37,38]. The solutions presented in [2,8,9,10,12,13,15,22,23] indicate that integration of the two disciplines is very promising for the development of sensors with excellent performance.

Besides outstanding properties in terms of sensitivity, accuracy, and resolution, to name a few, the sensors are sometimes required to have the capability of multiple detection, whilst preserving compactness. Most multiple-sensing devices are based on sensor arrays [30,31,35,37], which demand multiple outlets and separate read-outs, thus making the structure more complex. Although the microwave sensors in [5,16,17,18,19] are aimed at multiple sensing and employ one read-out system, such operation is achieved using multiple, physically well-separated sensing elements, leading to cumbersome structures.

One solution to this challenge would be the employment of a resonator that provides multiple, yet mutually independent resonant modes. Although various multi-mode microwave resonators have been proposed, such as resonators with perturbation, SIR and stub-loaded resonators [39], they do not provide entirely independent modes since there are no geometrical parameters that can independently control their even and odd modes.

In this paper, we present a compact microwave microfluidic sensor based on a dual-mode resonator with two independent resonant modes that can be used for concurrent sensing of two analytes without mutual influence. The resonator is comprised of a folded microstrip line loaded with a capacitor in the form of interdigital lines, and a stub at the plane of symmetry, which allows independent control of the fundamental odd and even modes, respectively. Dual-sensing capability is achieved by introducing two microfluidic channels—one in a manner to be intertwined with the interdigital lines and the other below the stub. Due to the multilayer configuration, the sensor has been fabricated using low-temperature co-fired ceramics technology (LTCC) and the laser micromachining process.

The potential of the proposed sensor has been demonstrated by measurement of its response, i.e., the spectral shift of the two resonances, for different fluids in the channels, whose dielectric constants range from 1, corresponding to air, to 80.1, corresponding to distilled water. It has been shown that the change of the properties of one fluid influences only one resonant mode, which confirms the dual-sensing potential of the sensor. Also, the sensor is characterized by good sensitivity, fast response, and compact dimensions. Moreover, with a slight modification of the microfluidic channels, the proposed configuration could be used as a micro-mixer in the precise mixing of fluids or for cell counting and sorting.

In the following sections, a detailed analytical and numerical analysis of the sensor will be presented, together with the description of the fabrication method and experimental setup. Simulated and measured responses will be given and the sensor’s performance discussed.

## 2. Sensor Design and Operating Principle

**Figure 1:**
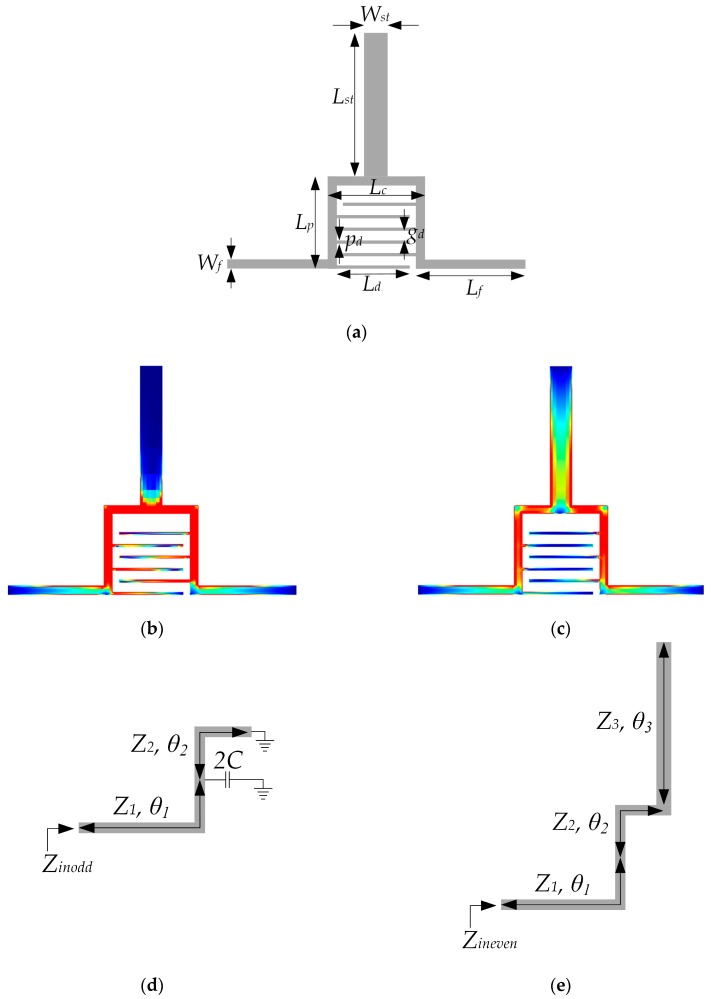
(**a**) Configuration of the proposed dual-mode resonator; (**b**) Current distribution for the odd mode; (**c**) Current distribution for the even mode; (**d**) Odd-mode equivalent circuit; (**e**) Even-mode equivalent circuit.

The core element of the proposed sensor is a dual-mode resonator whose layout and geometrical parameters are shown in Figure 1a. The resonator consists of a microstrip line loaded with a stub and interdigital lines at the symmetry plane. The microstrip line is folded to provide smaller overall size and, more importantly, to allow the capacitive coupling between the two segments of the length *L_p_* through the interdigital lines.

Due to the symmetry of the structure, the behavior of the resonator can be analyzed using even/odd-mode analysis. The current distributions of the two fundamental modes are shown in Figure 1b,c and they reveal that the first resonance is the fundamental odd mode resonance, whereas the second one is the fundamental even resonance. Equivalent odd- and even-circuits are shown in Figure 1d,e, where *θ*_1_, *θ*_2_, and *θ*_3_ represent the electrical lengths of the corresponding segments; *Z*_1_, *Z*_2_, and *Z*_3_ their characteristic impedances; whilst the parameter *C* represents the capacitance between the two segments of the length *L_p_*. One should note that *Z*_1_ is equal to *Z*_2_ due to the same microstrip line width. Also, *Z*_3_ is the characteristic impedance of the microstrip line whose width is half of that of the stub shown in Figure 1a. Counterintuitively *Z*_3_ is not equal to half of the characteristic impedance of the stub since microstrip characteristic impedance does not depend linearly on the line width.

Since the length and width of the area occupied by the interdigital lines are less than *λ_g_*/10 in the frequency range of interest, where *λ_g_* represents the guided wavelength, it can be considered that the interdigital lines represent a lumped capacitor and this will be further elaborated in the following sections. This is particularly important since one may argue that interdigital lines break the symmetry of the structure, which actually can be neglected due to their lumped nature.

Using the notation given in Figure 1d,e the input odd and even impedances can be expressed as:

(1) $$Z_{inodd}=Z_{1}\frac{\tan\theta_{2}-Z_{1}\tan\theta_{1}\tan\theta_{2}\omega 2C+\tan\theta_{1}}{j\left(Z_{1}\tan\theta_{2}\omega 2C-1+\tan\theta_{1}\tan\theta_{2}\right)}$$

(2) $$Z_{ineven}=Z_{1}\frac{Z_{3}-Z_{1}\tan\left(\theta_{1}+\theta_{2}\right)\tan\theta_{3}}{j\left(Z_{1}\tan\theta_{3}+Z_{3}\tan\left(\theta_{1}+\theta_{2}\right)\right)}$$

From the resonant condition 1/*Z_in_* = 0, the odd and even resonant mode equations are derived:

(3) $$Z_{1}\tan\theta_{2}\omega 2C-1+\tan\theta_{1}\tan\theta_{2}=0$$

(4) $$Z_{1}\tan\theta_{3}+Z_{3}\tan\left(\theta_{1}+\theta_{2}\right)=0$$

**Figure 2:**
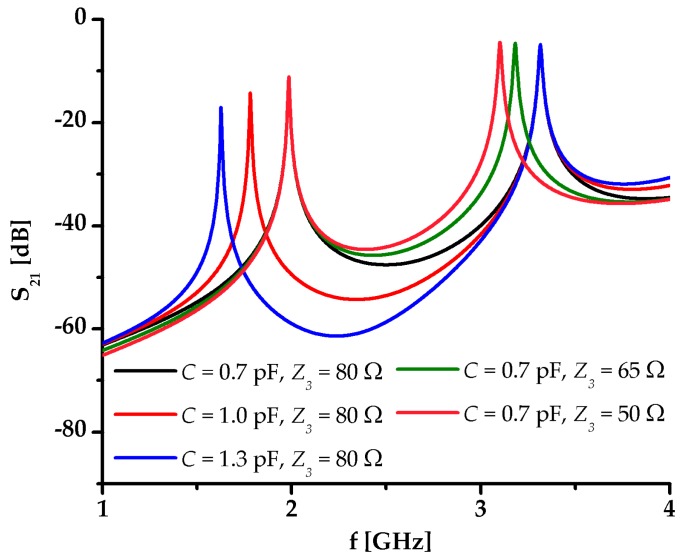
Response of the circuit model of the proposed resonator for different values of the capacitance of interdigital capacitor *C* and characteristic impedance of the microstrip line whose width is half of the stub *Z*_3_.

The expressions (3) and (4) reveal that the capacitance *C* affects only the odd mode, whilst the characteristic impedance and electrical length of the stub affect only the even mode. This is also confirmed in the circuit model response for different values of *C* and *Z*_3_, Figure 2. Such nature of the resonator, i.e., the two mechanisms for entirely independent control of the two resonant modes, opens up a possibility for the realization of a sensor with dual-detection operation, i.e., independent sensing of two analytes.

**Figure 3:**
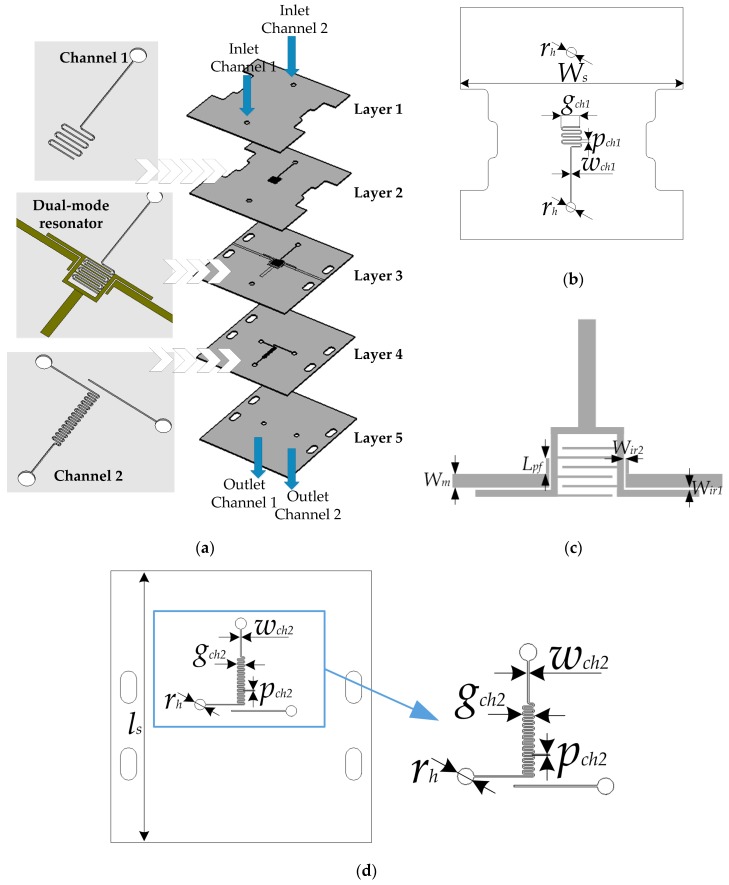
(**a**) 3D layout of the proposed sensor; (**b**) 2D layout of layer 2; (**c**) 2D layout of layer 3; (**d**) 2D layout of layer 4.

Figure 3 shows a 3D layout of the proposed sensor as well as 2D layouts of layers 1, 2, and 3. It can be seen that the sensor comprises the proposed resonator and two microfluidic channels positioned in the same layer with and below the resonator, resulting in a multilayer structure. Namely, the two microfluidic channels are introduced in the proposed microstrip structure to demonstrate the dual-sensing capability of the resonator. To achieve the best sensing performance, the positions of the microfluidic channels have to be judiciously chosen, taking into consideration the behavior of the resonator.

Since the first mode is independently controlled by the capacitance introduced by the interdigital lines, the most effective sensing is achieved if a microfluidic channel is meandered and occupies the area between the interdigital lines. On the other hand, the second mode is independently controlled by the stub’s characteristic impedance and electrical length, and therefore the most effective influence on the second mode is achieved if a microfluidic channel with the sensed fluid is positioned below the stub.

Layer 3 hosts the proposed dual-mode resonator together with the input microstrip lines. The resonator is capacitively coupled to the input lines primarily through the straight segments of the length *Lf*, to achieve a good transmission level of the signal. Although the coupling slightly influences the positions of the resonant modes, this can be neglected since the coupling equally affects the sensor’s response regardless of the sensed fluid.

Channel 1 is realized in three layers—2, 3, and 4—so it can be meandered in the area between the interdigital lines, and at the same time it does not intersect the conductive lines of the resonator nor does it have a contact with the interdigital lines. The microfluidic inlet and the meandered channel are made in layers 2 and 3, after which the fluid is transferred to layer 4 in which the channel outlet is realized. On the other hand, channel 2 is realized only in layer 4, since it is located below the stub and therefore does not need to span over several layers. Layers 1 and 5 serve as cover layers for the microfluidic channels and overall structure. Also, the small holes are used as inlet and outlets for the sensor, whilst the cuts in layers 1 and 2 and outermost holes in layers 3, 4, and 5 are made to allow the connectors to be assembled.

The proposed sensor features dual-sensing operation and both operating principles are based on the resonance concept, i.e., on the resonance shift caused by the change of the permittivity of the sensed fluid. Nevertheless, although resonance-based, the two operating principles differ in origin of the resonance shift.

As stated previously, the first resonance can be independently controlled by the change of the capacitance *C* that can be expressed as [40]:

(5) $$C=2\varepsilon_{0}\varepsilon_{effC}\frac{K\left(k\right)}{K^{\prime }\left(k\right)}\left(N-1\right)L_{d}\;\left(F\right)$$

where *N* represents the number of fingers, *L_d_* the length of each finger, *ε*_0_ air permittivity, and *ε_effC_* the effective permittivity of the microstrip capacitor. *K*(*k*) and *K’*(*k*) represent the elliptical integral of the first kind and its complement, respectively:

(6) $$\frac{K\left(k\right)}{K^{\prime }\left(k\right)}=\{\begin{array}{c} \frac{1}{\pi }\ln\{2\frac{1+\sqrt{k}}{1-\sqrt{k}}\}\;\mathrm{for}\;0.707\leq k\leq 1\\ \frac{\pi }{\ln\{2\frac{1+\sqrt{k^{\prime }}}{1-\sqrt{k^{\prime }}}\}}\;\mathrm{for}\;0\leq k\leq 0.707 \end{array}$$

(7) $$k=\tan^{2}\left(\frac{a\pi }{4b}\right),\;a=\frac{p_{d}}{2},\;b=\frac{p_{d}+g_{d}}{2},\;k^{\prime }=\sqrt{1-k^{2}}$$

The previous expressions show that the capacitance *C* can be varied by the change of the dielectric constant of the lower and/or upper dielectric layer, which indicate that the corresponding microfluidic channel can be positioned either above or below the interdigital lines to achieve sensing operation. Nevertheless, *C* also depends on the spacing between the lines, which can be intuitively comprehended since a pair of lines can be roughly considered as a miniature conventional parallel plate capacitor. Following these considerations, it can be concluded that the most effective sensing capability is achieved if the microfluidic channel is positioned “within” the interdigital lines, i.e., if it is realized as a meander that fills in the area between the lines. In that manner, the permittivity of the sensed fluid influences the capacitances of the miniature parallel plate capacitors, consequently effective permittivity of the lumped capacitor and overall capacitance *C*, and ultimately the position of the first resonance. It should be noted that once the microfluidic channels are positioned within interdigital lines, the previous expressions can be considered as approximate since the channels do not fill in the space between the capacitor fingers to the full extent.

**Figure 4:**
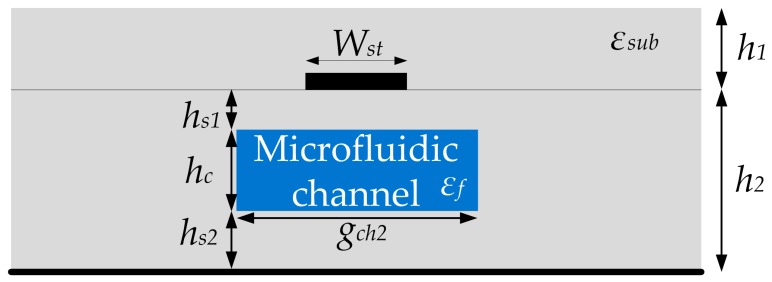
Cross-sectional view of the stub of the proposed resonator.

As for the second resonant mode, it was shown that it can be independently controlled by the electrical length and characteristic impedance of the stub, which depend on effective permittivity of the stub. Unlike the interdigital lines, the stub represents a conventional microstrip section whose effective dielectric constant can be most effectively varied by positioning the microfluidic line either above or below the stub. Since these two positions have practically the same effect on the stub, the channel is chosen to be positioned below the stub and thus the effective permittivity can be calculated if the structure in Figure 4 is considered. The top surrounding medium is made of LTCC dielectric substrate with the permittivity *ε_sub_*, whilst the bottom surrounding medium is made of the same material in which a microfluidic channel is embedded, having a dielectric constant that corresponds to the sensed fluid.

The effective permittivity of the stub can be approximated as:

(8) $$\varepsilon_{eff}=\frac{\varepsilon_{sub}+\varepsilon_{sf}}{2}+\frac{\varepsilon_{sub}-\varepsilon_{sf}}{2}\frac{1}{\sqrt{1+12\frac{h_{2}}{W_{st}}}}$$

where *ε_sub_* and *ε_sf_* are the permittivities of the upper dielectric and the bottom surrounding medium, respectively [41]. Since the bottom surrounding medium represents a combination of the LTCC dielectric material and fluid in the microfluidic channel, its effective permittivity can be expressed using the equation for the effective dielectric permittivity of the multi-layered substrate, [42]:

(9) $$\varepsilon_{sf}=\frac{\left|d_{1}\right|+\left|d_{2}\right|+\left|d_{3}\right|}{\left|\frac{d_{1}}{\varepsilon_{sub}}\right|+\left|\frac{d_{2}}{\varepsilon_{s+f}}\right|+\left|\frac{d_{3}}{\varepsilon_{sub}}\right|}$$

where the coefficients *d_n_* are:

(10) $$d_{1}=\frac{1}{\pi }\ln\left(2\frac{1+\sqrt{\frac{1}{\cosh\left(\frac{\pi W_{st}}{4h_{s1}}\right)}}}{1-\sqrt{\frac{1}{\cosh\left(\frac{\pi W_{st}}{4h_{s1}}\right)}}}\right)\;d_{2}=\frac{1}{\pi }\ln\left(2\frac{1+\sqrt{\frac{1}{\cosh\left(\frac{\pi W_{st}}{4\left(h_{s1}+h_{c}\right)}\right)}}}{1-\sqrt{\frac{1}{\cosh\left(\frac{\pi W_{st}}{4\left(h_{s1}+h_{c}\right)}\right)}}}\right)\;d_{3}=\frac{1}{\pi }\ln\left(2\frac{1+\sqrt{\frac{1}{\cosh\left(\frac{\pi W_{st}}{4h_{2}}\right)}}}{1-\sqrt{\frac{1}{\cosh\left(\frac{\pi W_{st}}{4h_{2}}\right)}}}\right)$$

The *ε_s+f_* is the permittivity of the middle layer with the channel that can be calculated using Bruggeman formalism [43]:

(11) $$\varepsilon_{s+f}=V\varepsilon_{s}+\left(1-V\right)\varepsilon_{f}$$

where *V* is the volumetric fraction of the microfluidic channel in the surrounding LTCC dielectric material, and *ε_f_* the permittivity of the fluid in the microfluidic channel.

Based on these expressions, the characteristic impedance and electrical length of the stub can be expressed as:

(12) $$Z_{stub}=\frac{60}{\sqrt{\varepsilon_{eff}}}\ln\left(8\frac{h_{2}}{W_{st}}+0.25\frac{W_{st}}{h_{2}}\right)$$

(13) $$\theta_{3}=L_{st}\frac{2\pi }{\lambda_{0}}\sqrt{\varepsilon_{eff}}$$

where *λ*_0_ is the wavelength in air.

The previous expressions reveal that the origin of the resonance shift of the second resonance is the change of the effective permittivity *ε_eff_*, which consequently changes the electrical length and characteristic impedance of the stub, and ultimately shifts the second resonance. Therefore, the resonance-shift principles in the case of the two resonances are somehow different since the first one is achieved through the capacitive effect, and the second one through the change of characteristic impedance and electrical length.

## 3. Simulation Results

The characteristics and the potential of the proposed sensor were first analyzed in numerical simulations run in CST Microwave Studio. Due to the multilayer configuration of the sensor, LTCC technology has been chosen for the fabrication process, and thus Ceramtec GC green tape with the following parameters was used as the substrate in simulations: *ε_r_* = 6.8 and tan δ = 0.002. Since the thickness of the green tapes is 0.3 mm, and their firing shrinkage around 18.5%, the thickness of each layer has been chosen to be equal to the integer number of the green tape’s thickness after firing, i.e., the thickness of each layer has been chosen to be 0.26 mm. In that manner, good stability in the firing process is provided as well as a strong coupling between the microfluidic channels and the corresponding parts of the resonator.

The geometrical parameters shown in Figure 1a and Figure 3 have been optimized to provide that the two resonances are spectrally sufficiently separated. In the case when the microfluidic channels are filled with air, the resonant frequencies are 2.02 and 3.34 GHz, respectively. The values of the parameters have been determined as follows: *W_st_* = 1.15 mm, *L_st_* = 7 mm, *L_p_* = 4.5 mm, *L_c_* = 4.75 mm, *p_d_* = 0.15 mm, *g_d_* = 0.45 mm, *W_f_* = 0.45 mm, *L_d_* = 3.55 mm, *L_f_* = 5.35 mm, *r_h_* = 2 mm, *W_s_* = 40 mm, *g_ch_*_1_ = 3.75 mm, *p_ch_*_1_ = 0.3 mm, *w_ch_*_1_ = 0.15 mm, *W_m_* = 1.2 mm, *L_pf_* = 1.35 mm, *W_ir_*_1_ = 0.2 mm, *W_ir_*_2_ = 0.15 mm, *g_ch_*_2_ = 1.25 mm, *p_ch_*_2_ = 0.16 mm, *w_ch_*_2_ = 0.16 mm and *l_s_* = 50 mm.

We would like to note at this point, that the provided values confirm that the interdigital lines comprise a capacitive structure whose dimensions are less than *λ_g_*/10, in the whole frequency range of interest, 1–4 GHz, regardless of the sensed fluid, and thus it can be considered as a lumped element.

**Figure 5:**
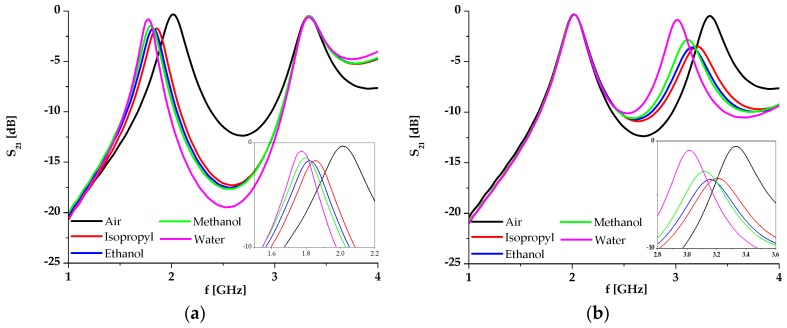
(**a**) Simulation results of the proposed sensor with different fluids placed in channel 1, whilst channel 2 is filled with air; (**b**) Simulation results of the proposed sensor with different fluids placed in channel 2, whilst channel 1 is filled with air.

The simulation results of the proposed sensor with different fluids placed in channel 1 and channel 2 when the other one is filled with air, are shown in Figure 5a,b, respectively. Each fluid in the simulation is modelled with its material parameters, i.e., its permittivity and dissipation factor, as shown in Table 1 [44].

The responses show that the variation of the analyzed fluid in one channel does not influence the response in the other resonance, which is clear proof of the dual-sensing capability of the sensor. The change of the fluid permittivity from 1 (air) to 80.1 (distilled water) causes the frequency to shift from 2.02 to 1.78 GHz in the case of channel 1, and from 3.33 to 3 GHz in the case of channel 2. It should be noted that changes of the resonance and loss in the transmitted signal depend on the material in the microfluidic channels. Since the changes are equal to 12% and 10%, the sensitivity of the proposed sensor is very good, which will be further discussed in the following section.

## 4. Fabrication and Measurement Results

**Figure 6:**
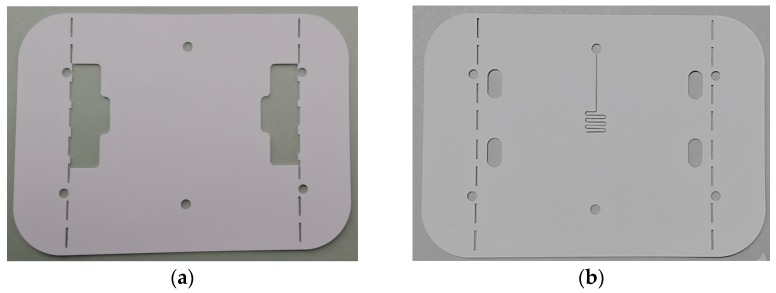

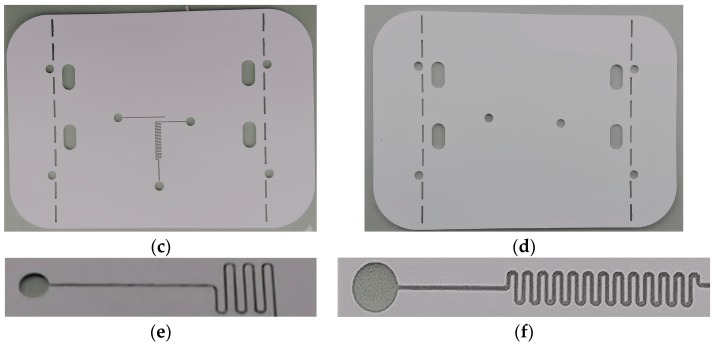
Green tapes after the cutting process: (**a**) Layer 1; (**b**) Layer 2; (**c**) Layer 4; (**d**) Layer 5; (**e**) Enlarged detail of layer 2; (**f**) Enlarged detail of layer 4.

**Figure 7:**
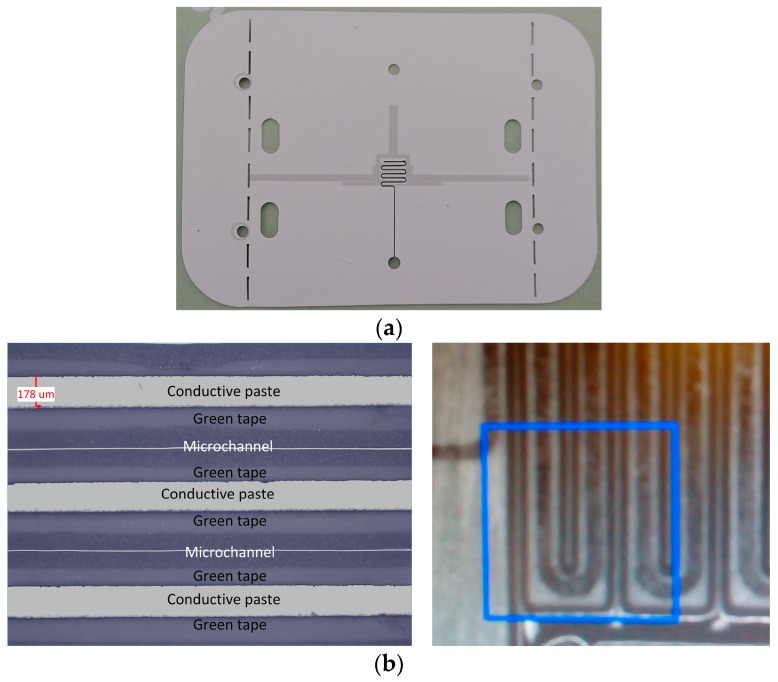
(**a**) Layer 3 after the cutting and screen printing processes; (**b**) Details of the interdigital capacitor with the meandered microfluidic channel in layer 3.

**Figure 8:**
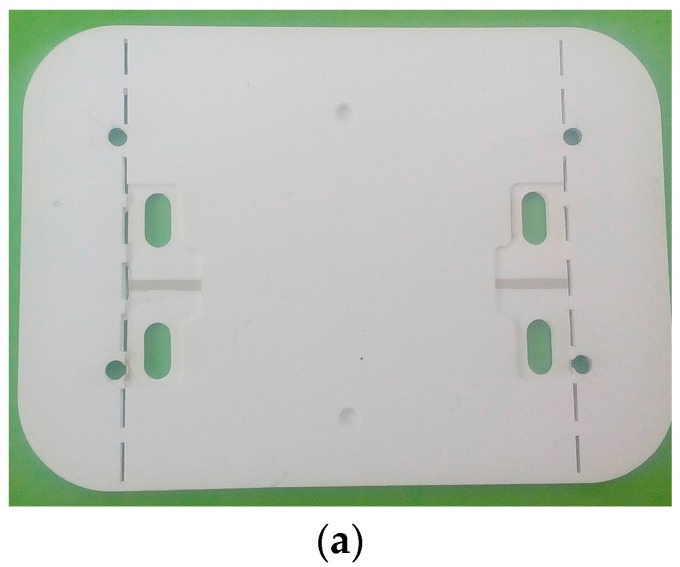

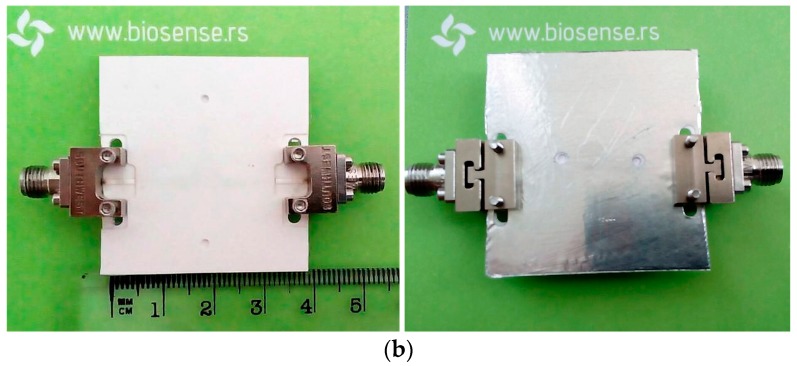
(**a**) The layout of the sensor after the lamination process; (**b**) The layout of the fabricated sensor—top and bottom view.

The proposed sensor has been fabricated using LTCC technology and a laser micromachining process. The fabrication process started with the preparation and precise cutting of the LTCC dielectric tapes with the aim to make the microfluidic channels, the holes for the inlet and outlets, and the cuts and holes for the connectors. Figure 6 shows the tapes after the cutting process carried out using Rofin-Sinar PowerLine D-100 laser, in which the sensor’s elements shown in Figure 3 can be observed. Figure 6e,f show enlarged details of the microfluidic channels cut in layers 2 and 4, respectively. The resonator and input and output microstrip lines were printed with the metal paste Heraeus TC7303 in the screen printing process using the EKRA M2H screen printer. Prior to the metal printing, a photomask had been prepared using UV exposure of the photosensitive film in the Technigraf Variocop S unit. Figure 7a shows layer 3 after the cutting and screen printing processes, whilst Figure 7b shows details of the interdigital capacitor with the meandered microfluidic channel, obtained with optical profilometer Huvitz HRM-300. It can be seen that the desired dimensions of the conductive lines and microfluidic channel have been obtained in the steps of cutting and screen printing. Once cut and printed, the layers were laminated, Figure 8a, and afterwards fired in the controlled firing process using the Nobertherm P330 furnace. After cooling of the circuits, 40 µm thick conductive aluminium sticky tape, cut with the laser, was added at the bottom of the sensor as a ground layer. In the final step, the connectors SMA Southwest Microwave 292-04A-5 were assembled to the circuit and Figure 8b shows the top and bottom views of the fabricated sensor.

**Figure 9:**
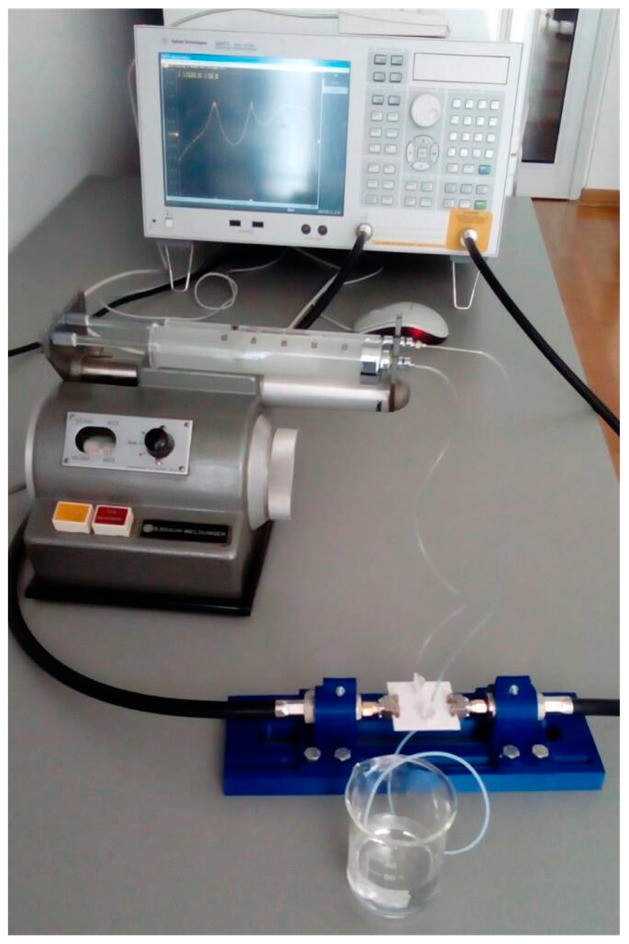
Measurement setup for the proposed sensor.

The measurement setup is shown in Figure 9, in which the fluids were injected through the additional tubules into the microfluidic channel using a syringe pump. To measure the responses for various fluids in each channel, the fluids were injected in one channel whilst the other one was kept empty, i.e., filled with air. The characteristic parameters of the fabricated sensor were measured in the frequency range between 1 and 4 GHz using a two-port Agilent 8501C Vector Network Analyser.

**Figure 10:**
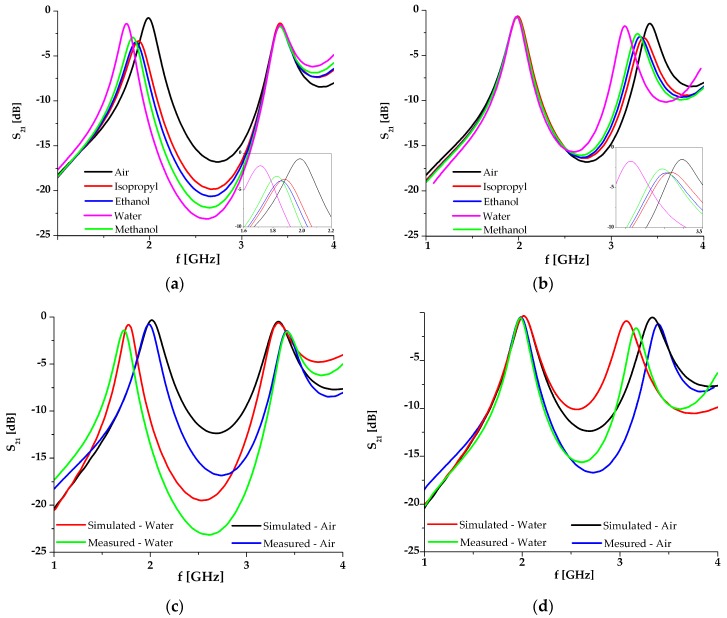
(**a**) Measurement results of the proposed sensor with different fluids placed in channel 1, whilst channel 2 is filled with air; (**b**) Measurement results of the proposed sensor with different fluids placed in channel 2, whilst channel 1 is filled with air; (**c**) Comparison of the simulation and measurement results for channel 1; (**d**) Comparison of the simulation and measurement results for channel 2.

The measurement results for different fluids in channels 1 and 2 are given in Figure 10a,b, respectively. The simulation and the measurement results are compared in Figure 10c,d. For better visibility, the measurements and simulations are compared only for the two fluids with the lowest and highest dielectric constant, i.e., air and water. It can be seen that the simulated and measured results are in good agreement, except for a small frequency shift which can be attributed to the fabrication tolerances. The measured results exhibit somewhat higher losses, which are however less than 4.4 dB, i.e., they are sufficiently good for this type of circuit.

**Figure 11:**
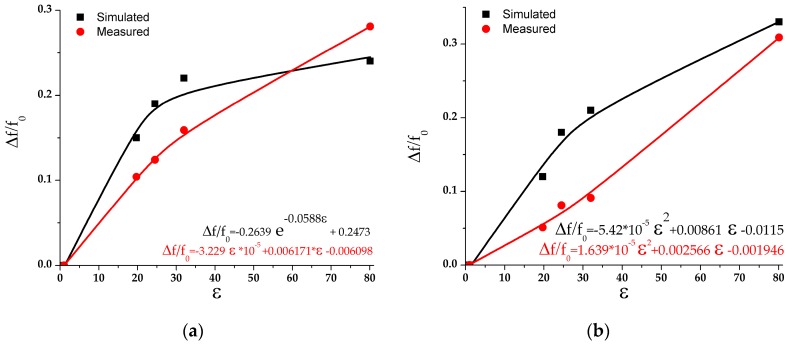
Frequency change (Δ*f*/*f*_0_) versus the change of fluid permittivity for: (**a**) Channel 1; (**b**) Channel 2.

Figure 11a,b show the frequency change (Δ*f*/*f*_0_) with respect to the change of fluid permittivity for channels 1 and 2, respectively, where *f*_0_ represents the resonant frequency when a channel is empty. Also, both figures include the graphs obtained from simulation and measured results as well as the fitting curves and corresponding equations obtained using the Matlab Curve Fitting Tool. The curves and equations for the measured results indicate that the proposed sensor shows almost linear dependence in both bands with a slight bend in the vicinity of the point *ε* = 30. Moreover, the measured results show a better linearity and a more stable trend than the simulated ones.

The proposed sensor is compared to other recently published microwave microfluidic sensors whose operating principle is based on resonance shift, Table 2. The comparison is made according to the overall circuit size and sensitivity. The circuit size is expressed in *λ_g_* that represents the guided wavelength at the resonance obtained for an empty channel, and in the case of the proposed resonator *λ_g_* is calculated for the first resonance. The sensitivity is defined as (*f_max_*–*f_min_*)/(*ε_max_*–*ε_min_*), where the parameters *f_min_* and *f_max_* correspond to the resonance positions for the cases of highest measured permittivity *ε_max_* and lowest measured permittivity *ε_min_*.

It could be seen that the proposed sensor has comparable size and sensitivity with the other sensors. The sensors proposed in [6,8] have significantly higher sensitivity, however it should be noted that they operate at frequencies higher than 10 GHz and thus the absolute resonance shift is more pronounced in those cases.

Besides comparable performance, the major advantage of the proposed microwave microfluidic sensor is the capability of dual-sensing, i.e., the possibility of simultaneous sensing of two analytes with zero mutual influence and one read-out system. Moreover, the proposed sensor represents a proof-of-concept and it can be further improved in terms of size and sensitivity. Namely, the resonator size can be reduced by the meandering of the stub or employment of fractal shapes to the microstrip segments. On the other hand, sensitivity can be improved if more interdigital lines with tighter distribution of the lines and channel are employed, and/or if the stub is widened and the channel accordingly extended.

Another important property of the proposed structure is the fact that it can find another application(s) in the field of microfluidics. Namely, if the configuration is slightly changed, the structure can be used as a micro-mixer in precise mixing of fluids or for cell counting and sorting.

## 5. Conclusions

A microwave microfluidic sensor with dual-sensing capability has been proposed. The sensor is based on a dual-mode resonator comprised of a folded microstrip line loaded with interdigital lines and stub. Owing to the specific configuration, the resonator exhibits two independent resonant modes and thus allows for simultaneous sensing of two analytes with one read-out system. To demonstrate the potential of the configuration, we have designed and fabricated a multilayer sensor with the proposed resonator and two separate microfluidic channels—one meandered and occupying the area between the interdigital lines and the other positioned below the stub. The sensors’ response for different fluids in the channels have been measured and the results confirmed dual-sensing capability, zero mutual influence as well as comparable size and sensitivity in comparison to other published microwave microfluidic sensors. Also, its performance can be further improved by optimization of the geometry of the structure. The proposed sensor has a significant potential not only for dual-sensing applications, but also for other microfluidics applications such as mixing fluids and cell counting.

## Figures and Tables

**Table 1:** Dielectric properties of the analysed fluids at room temperature.

Fluid	*ε_r_*	tanδ
Isopropyl	19.7	0.799
Ethanol	24.5	0.941
Methanol	32	0.659
Water	80.1	0.123

**Table 2:** Comparison of the characteristics of the proposed sensor and other recently published microwave microfluidic sensors with resonance shift operating principle.

Reference	*f_min_* (GHz)	*f_max_* (GHz)	*ε_min_*	*ε_max_*	Sensitivity (MHz/Relative Epsilon)	Overall Size (*λ_g_* × *λ_g_*)
[6]	14.05	17.08	1	80.1	38.35	0.5 × 0.5
[8]	8.9	10.5	1	80.1	20.25	5.8 × 5.8
[9]	3.4	4.2	1	80.1	10.13	0.23 × 0.22
[10]	0.915	1	1	80.1	1.07	0.19 × 0.08
[15]	1.57	1.875	16.5	72	5.5	0.1 × 0.1
[23]	1.18	1.52	1	80.1	4.3	0.086 × 0.094
This work resonance 1	1.703	1.984	1	80.1	3.57	0.21 × 0.17
This work resonance 2	3.072	3.381	1	80.1	3.91	
